# Supplementary material for: Shared and distinct microRNA profiles between HT22, N2A and SH-SY5Y cell lines and primary mouse hippocampal neurons
Source: PLoS One. 2025 Dec 3;20(12):e0326401. doi: 10.1371/journal.pone.0326401 (PMC12674520; doi:10.1371/journal.pone.0326401)
Supplement: S1 Fig — (PDF) [file pone.0326401.s001.pdf]

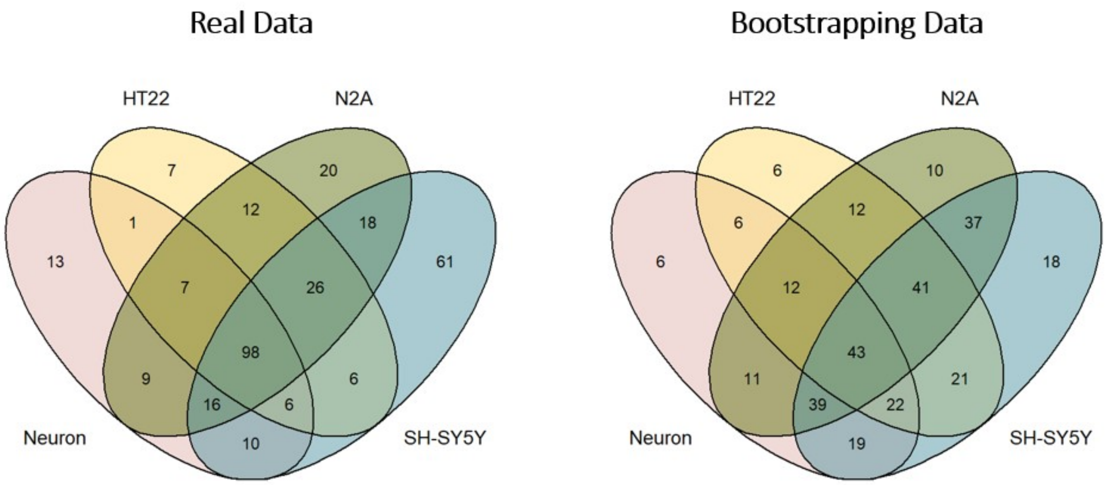

Supplementary Figure 1: Venn diagrams represent the overlap between the number of microRNAs expressed in our experiment (left, same as in Figure 2C) and the median number of microRNAs expressed by chance after 1000 bootstrapping iterations (right).
