# Supplementary material for: Shared and distinct microRNA profiles between HT22, N2A and SH-SY5Y cell lines and primary mouse hippocampal neurons
Source: PLoS One. 2025 Dec 3;20(12):e0326401. doi: 10.1371/journal.pone.0326401 (PMC12674520; doi:10.1371/journal.pone.0326401)
Supplement: S2 Fig — (PDF) [file pone.0326401.s002.pdf]

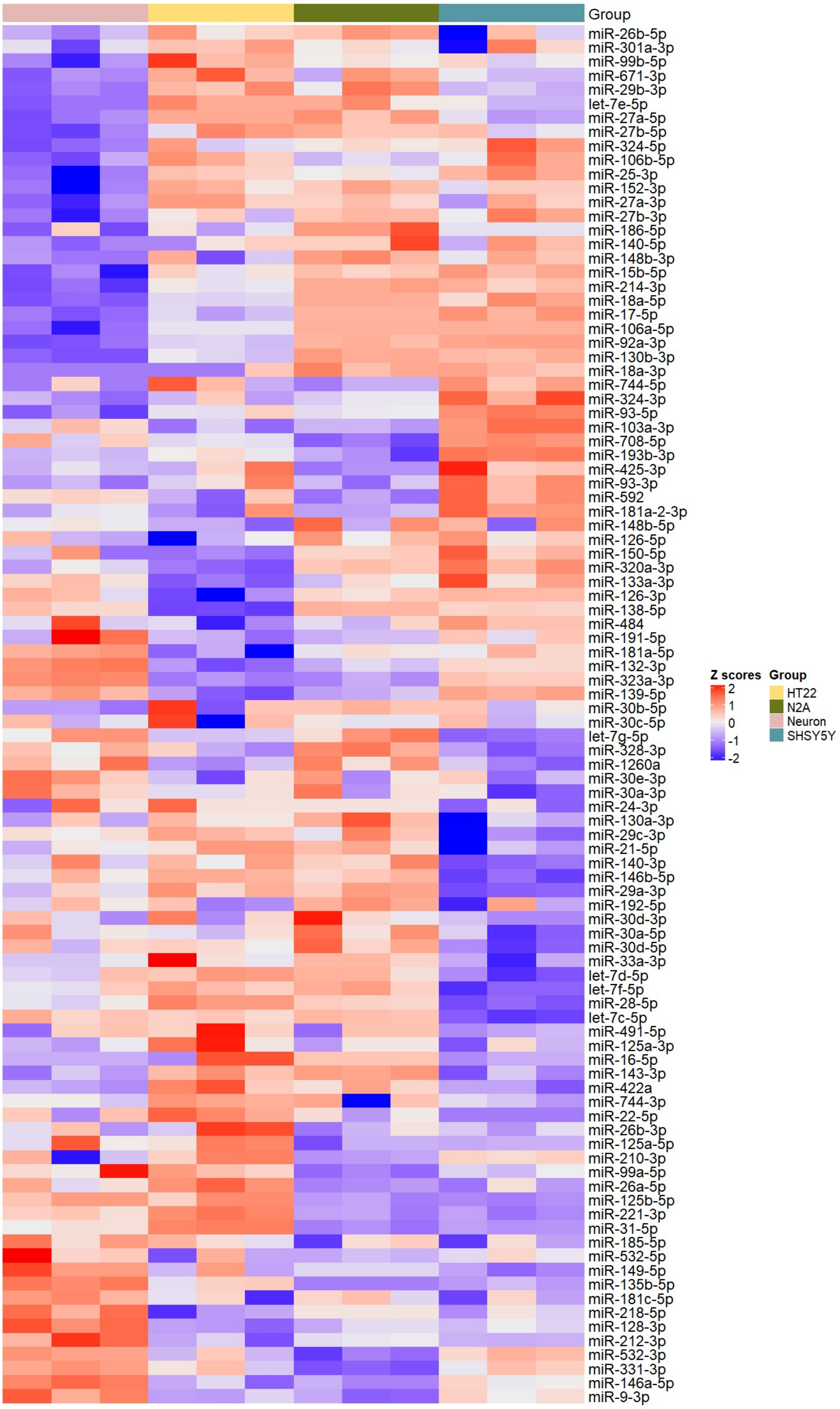

Supplementary Figure 2: Z-score Heat Map of the 98 microRNAs expressed in all four cell types, each line represents an independent microRNA.
