# Supplementary material for: Shared and distinct microRNA profiles between HT22, N2A and SH-SY5Y cell lines and primary mouse hippocampal neurons
Source: PLoS One. 2025 Dec 3;20(12):e0326401. doi: 10.1371/journal.pone.0326401 (PMC12674520; doi:10.1371/journal.pone.0326401)
Supplement: S3 Fig — (PDF) [file pone.0326401.s003.pdf]

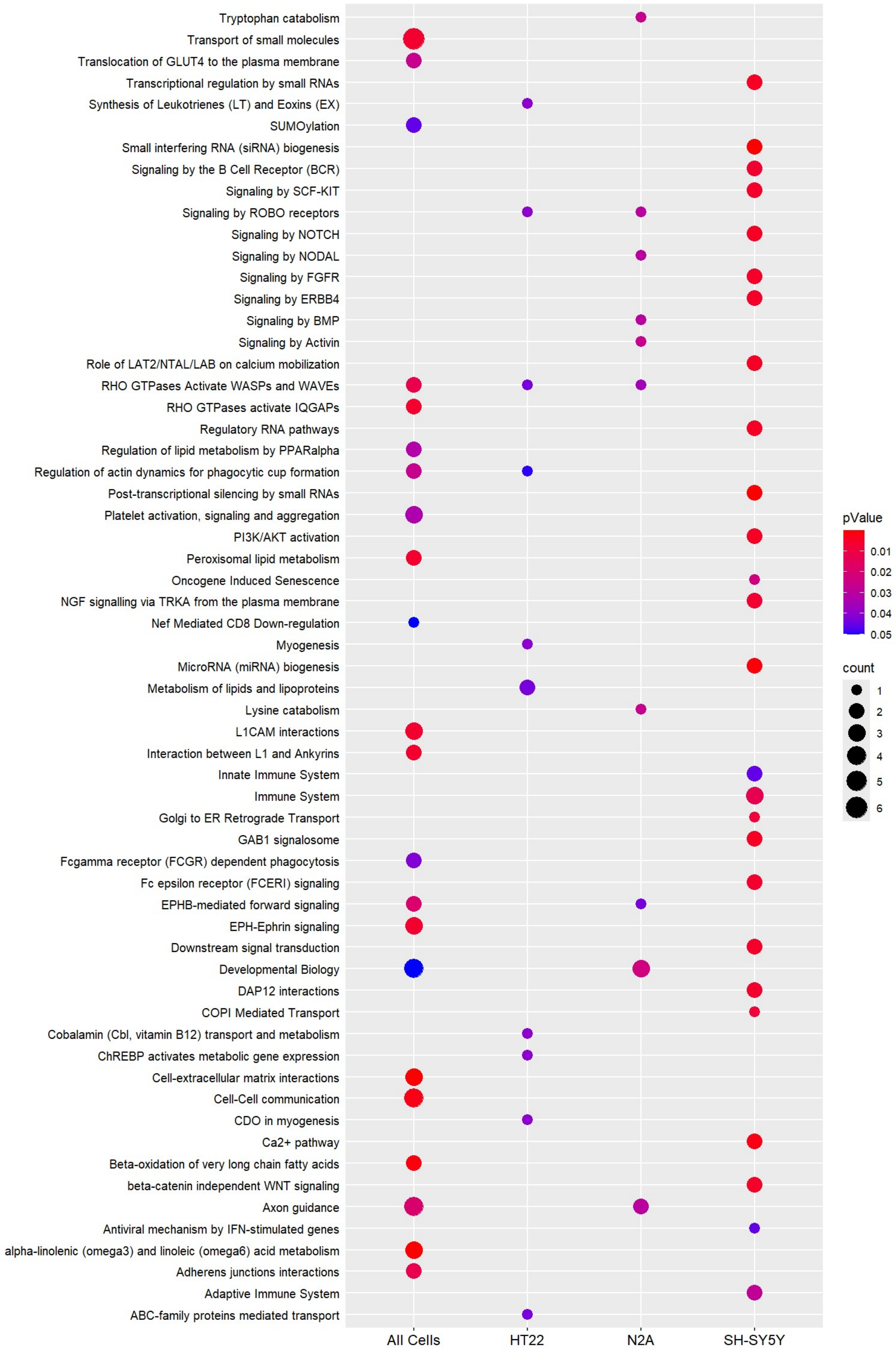

Supplementary Figure 3: Dot plot illustrating the enriched biological pathways of the microRNAs commonly expressed in all cell lines (left), and commonly expressed between primary hippocampal neurons and HT22, N2A and SHSY5Y (in order from left to right). The size of the dot represents the number of microRNAs involved and the colour codes the adjusted p-value.
