# Supplementary material for: Shared and distinct microRNA profiles between HT22, N2A and SH-SY5Y cell lines and primary mouse hippocampal neurons
Source: PLoS One. 2025 Dec 3;20(12):e0326401. doi: 10.1371/journal.pone.0326401 (PMC12674520; doi:10.1371/journal.pone.0326401)
Supplement: S1 Methods — (PDF) [file pone.0326401.s005.pdf]

Supplementary methods.

Bootstrapping analysis for the evaluation of microRNA distribution by chance.

To explore whether the distribution of microRNAs in the different cell lines might be simply random/chance, we used a bootstrapping approach to randomly determine the microRNA profile in each condition. To achieve this, we took the 310 microRNAs expressed by any of the four cell types and generated a random binomial distribution for each cell type using rbinom in R, with probability equal to the percentage of microRNAs expressed by that cell type in the original data. We performed 1000 iterations and compared the number of microRNAs in each condition (Supp. Figure 1). The histogram below shows for the number of microRNAs expressed by all four cell types in each bootstrapping iteration. The median number of commonly expressed miRNAs was 43 (95% CI = 32, 55). In our study, we observed 98 commonly expressed microRNAs, far above the upper range of the confidence interval. This suggests that the number of commonly expressed microRNAs is due to biological similarity rather than random chance. This approach was used for each of the conditions; the median value of each interaction was used to generate Supp. Figure 1.

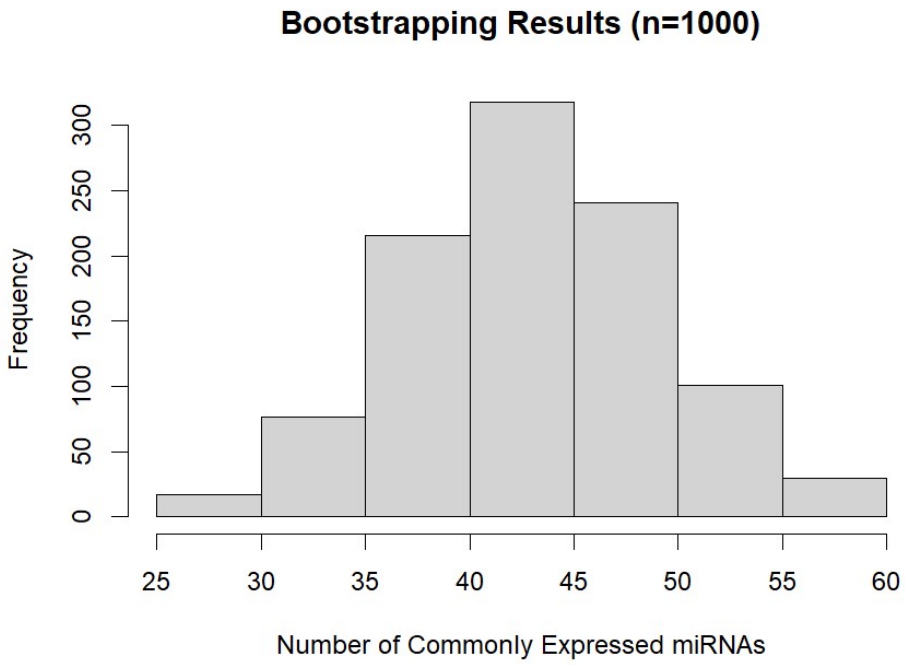

Histogram of the random distribution of microRNAs expressed by all cell types. The median number was used to generate the Venn diagram in Supplementary Figure 1.

Pathway analysis methods

Functional analysis was performed on all microRNAs that were similarly expressed in all 4 cell types, as well as on those uniquely similarly expressed between the primary neurons and each of the 3 cell lines, respectively. This analysis was performed using miRNet 2.0 (Chang et al., 2020). A network was created containing all experimentally validated miRNA-target interactions identified by miRTarBase 9.0 (Huang et al., 2022) and TarBase 9.0 (Skoufos et al., 2024). This network was filtered according to node degree (degree >= 1) and path length between nodes (shortest path between nodes). The Reactome pathways enriched by the remaining genes were identified using a hypergeometric test, with an adjusted p-value of < 0.05 considered significant.

Reference

Chang, L., Zhou, G., Soufan, O., Xia, J. (2020) miRNet 2.0: network-based visual analytics for miRNA functional analysis and systems biology, Nucleic Acids Research, 50(W1), W44–W251. https://doi.org/10.1093/nar/gkaa467

Huang, H. Y., Lin, Y. C., Cui, S., Huang, Y., Tang, Y., Xu, J., Bao, J., Li, Y., Wen, J., Zuo, H., Wang, W., Li, J., Ni, J., Ruan, Y., Li, L., Chen, Y., Xie, Y., Zhu, Z., Cai, X., Chen, X., ... Huang, H. D. (2022). miRTarBase update 2022: an informative resource for experimentally validated miRNA-target interactions. Nucleic acids research, 50(D1), D222–D230. https://doi.org/10.1093/nar/gkab1079

Skoufos, G., Kakoulidis, P., Tastsoglou, S., Zacharopoulou, E., Kotsira, V., Miliotis. M., Mavromati, G., Grigoriadis, D., Zioga, M., Velli, A., Koutou, J., Karagkouni, D., Stavropoulos, S., Kardaras F. S., Lifousi, A., Vavalou, E., Ovsepian, A., Skoulakis, A., Tasoulis, S. K., Georgakopoulos, S. V., Plagianakos V. P., Hatzigeorgiou, A. G. (2024) TarBase-v9.0 extends experimentally supported miRNA–gene interactions to cell-types and virally encoded miRNAs, Nucleic Acids Research, 52(D1), D304–D310. https://doi.org/10.1093/nar/gkad1071
