# Supplementary material for: Shared and distinct microRNA profiles between HT22, N2A and SH-SY5Y cell lines and primary mouse hippocampal neurons
Source: PLoS One. 2025 Dec 3;20(12):e0326401. doi: 10.1371/journal.pone.0326401 (PMC12674520; doi:10.1371/journal.pone.0326401)
Supplement: Attachment — Submitted filename: Response to reviews - Murphy et al..doc [file pone.0326401.s007.doc]

We would like to thank the reviewers for the comments that have helped to improve our manuscript. As requested by both reviewers, we have uploaded the raw data in the Supplementary Information as an Excel file. Please find below a detailed point-by-point discussion of the concerns and suggestions raised during the review process. Changes to the revised manuscript are highlighted in the text.

**Reviewer 1**: We thank the reviewer for their positive assessment of our manuscript. Please find below the response to each comment point-by-point:

1. **Please attach the raw data of the miRNA analysis.**

*Answer: The raw data has been made available in the Supplementary Information as an Excel file. This information has been updated in the manuscript under the Methods section, Analysis of Open Array subsection, page 8, lines 166-167.*

2. **Why hippocampus and a hippocampal cell line were used as a control, since only HT22 cells originate from the hippocampus?**

*Answer:* *We thank the reviewer for raising this point. As the reviewer correctly points out, HT22 is the only cell line with a hippocampal origin.* *The rationale for the inclusion of two additional [non-hippocampal] cell lines was two-fold. First, to provide context for the microRNA profile of the HT22 cell lines by comparing them to other immortalised cell lines (vs primary neurons). This enables the reader to better appreciate what signatures might reflect tissue of origin vs immortalisation. Second, the widespread use of the other two cell lines in neuroscience research makes their inclusion an obvious strength and point of general interest. Despite not having a hippocampal tissue origin, we nevertheless identified microRNA profiles that resembled primary neurons. This may provide assurance to researchers using these non-hippocampal lines that their findings will bear relevance to primary hippocampal neurons (in the event they choose not to switch to HT22 for other reasons).*

*We have clarified the value of including the other two cell lines in our revised manuscript in the Discussion section page 14, lines 302-37: “Importantly, the current study compared hippocampal primary neurons not only to the HT22 line which are of hippocampal origin, but also neuroblastoma cell lines N2A and SH-SY5Y. This adds important value to the present findings in two ways. First, the inclusion enables the identification of microRNA signatures that reflect tissue origin versus immortalisation. Second, the inclusion of these cell lines gives the fingerprint of the similarities between both neuroblastoma cell lines and primary hippocampal neurons”.*

3. **A bioethical statement for laboratory animal use is missing**

*Answer:* *All procedures were performed in accordance with the guidelines of the European Communities Council Directives (86/609/EU and 2010/63/EU) and were reviewed and approved by the Research Ethics Committee of the Royal College of Surgeons in Ireland (REC #765) under license from the Department of Health, Dublin, Ireland (B100/4524). We have updated this information on the Methods section, primary cell culture subsection, page 6, lines 111-114.*

4. **Were the cells authenticated and tested for mycoplasma?**

*Answer: All cell lines were tested for mycoplasmas every 6 months; cell lines used in those experiments tested negative for the presence of mycoplasmas. We have updated the cell culture subsection in the methods to contain this information as:* *“Cell lines were tested for mycoplasmas before experiments started. Results were negative for the three immortalized cell lines” (Methods section, cultured cell line subsection, page 7, lines 136-137)*

5. **Authors aimed to compare the expression profile of cells based on similarity using Pearson correlation analysis. This type of analysis is only a poor descriptor of similarity. I recommend using a different metric for this.**

*Answer: To validate the Pearson correlation analysis, we have used the Mean Squared Error as a measure of similarity. Using this approach our HT22 cell have a score of 8.515, N2A cells have a score of 13.444 and SH-SY5Y cells have a score of 8.443. This data strongly aligns with our previous analysis. This information has been added to the text in the methods section page 8, lines 158-159 (Correlation was calculated using the Pearson correlation coefficient and the Mean Squared Error to evaluate similarity), results section page 12, lines 255-257, and in the Figure 4 legend (Correlations between the expression levels of the individual microRNAs between the primary hippocampal neurons and A) HT22 (r=0.700, MSE=8.515); B) N2A (r=0.564, MSE=13.444); and C) SH-SY5Y (r=0.721, MSE=8.443).*

6. **Authors stated on several occasions percentages of overlapping miRNAs expressed in their cell line. While these numbers are interesting, they should be considered in comparison to the number expected by chance for the given number of expressed miRNA.**

*Answer: Assuming that all possibilities are equally likely, we have a total of 16 scenarios, A single microRNA can be expressed in all of the groups, none of the groups, exclusively expressed in one group or expressed in a selected number of groups (2 or 3 of them). Taking into consideration these scenarios and that these are equally possible, the chance of a microRNA expressed in all groups is 6.25%. This strongly supports that our profile is not due to a random chance.*

7. **Authors talked about the function of selected miRNA in their manuscript. I highly recommend the authors to do a functional association analysis (data base analysis) of all miRNA that are expressed jointly in all cell lines/primary cells and all those that differ between the cell lines. As authors propose, one main goal of the manuscript is to characterize these cell lines for potential further use cases it is of importance to narrow down the functional implications of differential and similar miRNA expression as much as possible.**

*Answer: Thank you for the comment. We have generated two new Figures (Supplementary Figure 2 and 3) to represent the enriched pathways regulated by the microRNAs expressed in all cell lines and primary hippocampal neurons (Supp. Figure 2), and unique to each cell type (Supp. Figure 3). Regarding the first figure, we have also added the enriched biological pathways regulated by the commonly expressed microRNAs between the primary hippocampal neurons and each of the immortalized cell lines as requested by Reviewer 2, Point 3.*

*We have included this information in the Results section pages 12-13, lines 272-283: “* *Then, we evaluated the biological pathways enriched in all cell lines combined, primary cell cultures and/or each cell line. Axon guidance, cell-to-cell communication, and transport of molecules were the most significantly regulated within the 98 common microRNAs expressed in all the cell types (Supp. Figure 2). Metabolism was the main pathway regulated by microRNAs expressed in primary hippocampal neurons and HT22. Axon guidance and developmental biology was found between primary hippocampal neurons and N2A. And at last, RNA regulation and post-transcriptional regulation, between primary hippocampal neurons and SH-SY5Y cells (Supp Figure 2). Finally, we evaluated the microRNAs-regulated pathways by the specific microRNAs expressed exclusively in each cell line. This revealed that pathways involved in Calcium regulation and CREB transcription factor signalling activation are only seen in the primary cell culture, while mTOR and S6K signalling pathways are only seen in HT22 cell line, extracellular matrix regulation is observed only in N2A cell lines, and, finally, in SH-SY5Y cell lines has pathways related to cytokine regulation and innate immune system (Supp. Figure. 3).”*

*Methodology for this section has been included in the supplementary information, under the subheading supplementary methods.*

8. **Figure 2D: Please use a different way to illustrate the data as it is hard to follow the individual expression of those miRNAs, e.g. as a heatmap of z-scores. Please provide the raw data for this figure as a supplemental table.**

*Answer: Thank you for the suggestion. We propose to retain the original Figure 2 since a key value is that it shows relative abundance of each microRNA in the different cell lines. However, we have generated a z-score heat map to complement the data in Figure 2. This has been included as supplementary information (Suppl Figure 1) and in the text page 10, line 219.*

9. **Fig 4 is blurred. Please exchange it**

*Answer: Thank you for the comment. We have uploaded a higher-quality image of Figure 4.*

10. **Table 1 is twice in the manuscript**

*Answer: Thank you for the comment. We have removed one of the copies of Table 1 from the manuscript.*

11. **How was the hippocampal tissue isolated, which mouse strain and how old were the mice?**

*Answer: For the primary hippocampal cell cultures, C57BL/6J pregnant females were obtained from the Biomedical Research Facilities (RCSI). Embryonic day 18 (E18) old pups were used for these experiments. Hippocampi were isolated and tissue dissociated using the papain protocol as previously described by Jimenez-Mateos et al., 2012. This information has been clarified in the Methods section, primary cell culture subsection, page 6, lines 116-124.*

12. **In Fig 1 legend several typos “tisuue” and “hypocampal” are present**

*Answer: Thank you for the comment. Typos have been corrected in the Figure legend, see page 19 lines 413 and 414, respectively.*

**Reviewer 2**: We thank the reviewer for their comments and suggestions. Please find below the response to each comment point-by-point:

1. **The minor comment concerns the use of tissue from epileptic patients. These samples are neither adequately referenced nor described in the Results and Discussion sections. Simply stating “human hippocampus” is not sufficient.**

*Answer: The data used in the current manuscript have been previously published in Mills et al., 2019 (Ref. 46 in manuscript). From this original manuscript, we used data from 3 hippocampal post-mortem control tissues after obtaining permission from the authors (see acknowledgement section). Control material was obtained during autopsy resection, and a history of no seizures or other* [*neurological diseases*](https://www.sciencedirect.com/topics/pharmacology-toxicology-and-pharmaceutical-science/neurologic-disease) *was confirmed.*

*To clarify this, we have revised the manuscript and have added the following information in the Methods section, Human data and RNA-Seq data analysis subsection page 8, lines 170-172: “**RNA-seq raw data was obtained from previously available data [46]. Hippocampal post-mortem tissue was obtained during autopsy resection and a history of no-seizures or other* [*neurological diseases*](https://www.sciencedirect.com/topics/pharmacology-toxicology-and-pharmaceutical-science/neurologic-disease) *was confirmed as described in the original publication [46].”*

**The three major comments are:**

**1. I could not find any supplementary material containing the primary data used in this study, which is essential to verify the analyses and the figures presented. Without access to these data, the results are not reproducible and cannot be readily reused by other researchers.**

*Answer: The raw data has been made available at Supplementary Information as an Excel file. This information has been updated in the manuscript under the Methods section, Analysis of Open Array subsection, page 8, lines 166-167.*

**2. The authors mention the lack of miRNA profiling in differentiated cells of the tested lines as a limitation. I believe this is a critical point for the study’s objectives. While I understand the technical and resource demands of such an experiment (and that it may be part of future work), a minimum step should be to compare neural stem cells with primary neurons. For this purpose, RNA could be extracted directly from mouse cortices at E11/E12 (the peak of neural stem cell proliferation), or alternatively cultured until E14 under proliferative conditions.**

**This comparison would be valuable for two reasons:**

**a) To determine whether there is a significant shift in miRNA profiles between neural progenitors and differentiated/mature neurons. It is also possible that the profiles remain largely similar.**

**b) Additionally, it would help in fitting the profiles obtained from the various cell lines along a continuum from progenitors to mature neurons (especially if a significant difference is observed in point a).**

**Two possible scenarios may emerge:**

**• If no significant difference is observed between progenitors and mature neurons, the conclusions of the study would shift toward identifying which lines most closely resemble general neural cells, rather than neurons specifically.**

**• If a clear difference exists, it would then be possible to classify each cell line as being more similar to either progenitors or mature neurons.**

*Answer: We thank the reviewer for this valuable point and suggesting to analyse neural progenitors as an additional comparator. Unfortunately, the PCR-based Open Array platform used to generate the profiles in the original study has notable batch-to-batch effects, which would make such a direct comparison problematic. We consider the use of previously available data; however, all open access data found by the authors on neural progenitor cells were not suitable for this study, as they were transgenic mice or treated with vehicles, such as ethanol or DMSO. Nevertheless, we consider this a good point, and we have added the following paragraph in the discussion (page 16, lines 365-369): “And last, in the current study, we have compared immortalized cell lines to mature hippocampal neurons, and not to neural progenitor cells. The limitation here, is the difference in maturation state and we cannot be certain whether the cell lines are more similar to progenitors or mature neurons. This could be explored in future experiments, comparing progenitors to varying states of maturation using primary hippocampal neurons”.*

**3. Functional analyses (e.g. miRNet 2.0 computational tool) should be performed on the differentially expressed miRNAs across the various comparisons shown in Figures 4 and 5. The current manuscript only highlights a few selected miRNAs, despite many others showing differential expression. On what statistical or biological criteria were these specific miRNAs chosen? This selection needs to be clarified and justified.**

*Answer: Thank you for the comment. We have generated a new Figure (Supplementary Figure 2) to represent the enriched biological pathways regulated by the commonly expressed microRNAs between the primary hippocampal neurons and the cell lines (Figure 4) and microRNA commonly expressed in all the cell lines and primary hippocampal neurons as requested by Reviewer 1 (see Reviewer 1, point 6).*

*We have included this information on the Results section pages 12-13, lines 272-283: “ Then, we evaluated the biological pathways enriched in all cell lines combined, primary cell cultures and/or each cell line. Axon guidance, cell-to-cell communication, and transport of molecules were the most significantly regulated within the 98 common microRNAs expressed in all the cell types (Supp. Figure 2). Metabolism was the main pathway regulated by microRNAs expressed in primary hippocampal neurons and HT22. Axon guidance and developmental biology was found between primary hippocampal neurons and N2A. And at last, RNA regulation and post-transcriptional regulation, between primary hippocampal neurons and SH-SY5Y cells (Supp Figure 2). Finally, we evaluated the microRNAs-regulated pathways by the specific microRNAs expressed exclusively in each cell line. This revealed that pathways involved in Calcium regulation and CREB transcription factor signalling activation are only seen in the primary cell culture, while mTOR and S6K signalling pathways are only seen in HT22 cell line, extracellular matrix regulation is observed only in N2A cell lines, and, finally, in SH-SY5Y cell lines has pathways related to cytokine regulation and innate immune system (Supp. Figure. 3).”*

*On figure 5, we decided to show a representation of microRNAs to emphasize the similarities between primary hippocampal neurons and the cell lines. For this, we focused on microRNAs linked to neuronal function. This has been added for clarification on the Results section page 12 lines 285-286:” particularly those microRNAs previously linked to neuronal function.”*

*Methodology for this section has been included in the supplementary information, under the subheading supplementary methods.*
