# Supplementary material for: Shared and distinct microRNA profiles between HT22, N2A and SH-SY5Y cell lines and primary mouse hippocampal neurons
Source: PLoS One. 2025 Dec 3;20(12):e0326401. doi: 10.1371/journal.pone.0326401 (PMC12674520; doi:10.1371/journal.pone.0326401)
Supplement: Attachment — Submitted filename: Second Response to reviews - Murphy et al..doc [file pone.0326401.s008.doc]

We would like to thank the reviewer for the further comments that have helped to improve our manuscript.

**Reviewer 1**: We thank the reviewer for their positive assessment of our manuscript. Please find below the response to each comment point-by-point:

1. **Point 6: When calculating the possibility for the overlap to be caused by chance, the approach of the authors is sub-par, as it does not include the proportion of expressed/differently expressed (depending on the context) miRNAs relative to the total amount of miRNAs tested for. As a thought experiment to illustrate this idea imagine a tumor cell line expressing all tested miRNAs and comparing them to any cell line. Thus, this analysis would deem them highly similar and would fail to identify the result as being caused by chance. Consequently, another estimator to account for randomness is needed. A typical approach could be based on bootstrapping. E.g. authors could calculate the percentage of miRNAs expressed or over-expressed (depending on the context) in one of the samples to be compared, and than randomly resample the miRNAs expressed/over-expressed to create a virtual new data-set and now check the overlap between the random set and the data set to be compared to . This corresponds to one bootstrapping iteration. This procedure needs to be repeated at least 1000 times to obtain 1000 values for the percantage of overlap. The mean value of these 1000 percentages would be a descent estimate for the probability for the amount of overlap expected due to chance alone, independent on biological similarity.**

*Answer: Thank you for the comment. We have now run a Bootstrapping Re-sampling analysis to account for random distribution. We have generated a new Supplementary Figure 1. Supp. Figure 1 shows side-by-side the distribution of microRNAs in our experiment and compares it to the Resampling set. We have update the information on page 8 line 168-172: “*Further analysis was carried out to evaluate if the distribution of the number of microRNAs expressed in each condition is due to chance using the Bootstrapping resampling method. Bootstrapping iterations were performed using random binomial resampling with probability equal to the percentage of microRNAs expressed by each cell type in the original data (see Supp. Figure 1 and methods)”. *On page 10 lines 221-225:*”To determine whether the observed overlap in miRs between the different cell lines was due to random chance, we performed 1000 bootstrapping iterations and compared the median results to our original data (Supp. Figure 1 and methods). This revealed that the resampled distribution was significantly different from the distribution in our profile (Supp. Figure 1), and thus the overlap in miR expression across the cell lines is likely due to biological similarity rather than random chance.”. *And we have added the new methods to the Supplementary data section*:

“ Bootstrapping analysis for the evaluation of microRNA distribution by chance.

To explore whether the distribution of microRNAs in the different cell lines might be simply random/chance, we used a bootstrapping approach to randomly determine the microRNA profile in each condition. To achieve this, we took the 310 microRNAs expressed by any of the four cell types and generated a random binomial distribution for each cell type using rbinom in R, with probability equal to the percentage of microRNAs expressed by that cell type in the original data. We performed 1000 iterations and compared the number of microRNAs in each condition (Supp. Figure 1). Supp. Figure 5 shows the histogram for the number of microRNAs expressed by all four cell types in each bootstrapping iteration. The median number of commonly expressed miRNAs was 43 (95% CI = 32, 55). In our study, we observed 98 commonly expressed microRNAs, far above the upper range of the confidence interval. This suggests that the number of commonly expressed microRNAs is due to biological similarity rather than random chance. This approach was used for each of the conditions; the median value of each interaction was used to generate Supp. Figure 1.”

**Reviewer 2**: We thanks Reviewer 2 for the minor comments/suggestions. See our response below point by point :
**1. Line 56, modify "Argonuate" in "Argonaute"**

*Answer:* *We have corrected this typo.*

**2. Line 170, I suggest to modify in "****RNA-seq raw data of hippocampus of control human donor was obtained from previously available data []. Hippocampal post-mortem tissue ..."**

*Answer:* *The line has been corrected following the reviewer’s recommendation (Line 175).*
